# Surgery

**Published:** 1906-06

**Authors:** Charles A. Morton


### SURGERY.

Although so much is written at the present time on appendicitis and its sequelæ that I rather hesitate to refer to it again in this periscope, yet what is written on the subject by a surgeon who has operated on 2,000 cases is likely to be of considerable value. Murphy, of Chicago, well known in connection with his button, published a paper in the *American Journal of Medical Sciences* for August, 1904, on **Two Thousand Cases of Operation for Appendicitis.** Six hundred operations were for acute appendicitis, and about 1,400 after the subsidence of the attack.

With regard to the symptoms of the acute attack, he says they always occur in the following order :—Pain ; nausea and vomiting ; general abdominal sensitiveness, most marked on the right side, and more particularly over the appendix ; pyrexia, beginning from two to twenty-four hours after the commencement of the pain. He says if the nausea or vomiting, or temperature precede the pain he feels sure the case is not one of appendicitis, and that temperature before pain suggests typhoid fever with a typhoid ulcer in the appendix. He has operated on three such cases, and has seen four more which he believed to be of the same nature. "Sensitiveness" preceding the other symptoms, especially if low in the pelvis, he considers suggestive of infection of the Fallopian tube, or of tuberculous peritonitis, rather than appendicitis. Pain, he says, is never absent as an initial symptom in appendicitis. He states that there is always some rise of temperature during the first thirty-six hours, and that this may occur during the first few hours or not for twenty-four. If he were confident that no rise of temperature had been present during the first thirty-six hours, he says he should not regard the case as one of appendicitis. But he also says the temperature may fall to normal again within twenty-four hours; so that it would be very seldom possible to say there had certainly been no temperature at any time within the first thirty-six hours unless an hourly chart had been kept. In any case of doubtful diagnosis it might be a help to get an hourly chart for the first thirty-six hours if possible. His experience, as well as that of other surgeons, has been that the temperature may be high, and then fall below 99° and remain there, and yet a large quantity of pus may be present. He refers to one case in which the temperature suddenly dropped from 104° to 99°, and on opening the abdomen at the time the whole appendix was found to be gangrenous and without any protecting adhesions. He refers to the apparent presence of a swelling, the size of the thumb, in the appendix region "in the very early hours" of appendicitis, due to muscular contraction, which disappears under the anæsthetic. Such apparent swelling may be thought to be the swollen appendix. He thinks the most difficult condition to distinguish from appendicitis is an acute catarrhal affection of the caput coli, especially "associated with the intestinal form of *la grippe*." He operated twice in this condition, expecting to find acute appendicitis, but only found evidence of old attacks, and he says he knows no means of distinguishing such cases from appendicitis.

He maintains that it is impossible to tell in the first forty-eight hours whether the tendency will be towards subsidence or to a virulent, if not fatal, termination, and this is why he operates on all cases; and he says the danger of operation in the early stage is scarcely more than that of an exploratory laparotomy. He thinks that at this stage drainage is not as a rule indicated, and therefore hernia improbable. He says the patient would be relieved of his appendicitis without hazard, prolonged illness, the danger of unpleasant sequelæ, or the possibility of recurrence by what he regards as "the only timely operation." He refers to a case in which thirty-one hours after the onset of the pain the pulse had fallen from 110 to 82, and the temperature from 102.8° to 100.2°; but on the abdomen being opened a circular gangrene of the appendix one-half inch in length was found. He points out that we cannot tell what the tendency of the pathological process may be, whether towards security and repair or towards destruction and danger. Unfortunately, with regard to the nature of the operation to be performed in what he calls the second stage, *i.e.* from the second to the fifth day, he is neither clear nor consistent, but that we should operate he is emphatic. He first says that the operation must be a limited one, consisting of simple relief of pus tension by drainage, and that the appendix is only to be removed if it is accessible and easily amputated, and that there should be the least possible separation of adhesions, which are life-saving both in circumscribing the process and in rendering the local tissue unfit for absorption; and again further on he says we must never lose sight of the fact that the pus is usually virulent, and that when organised adhesions are present they must not be separated, as they expose the surface to acute absorption. And yet he says "in the ascending stage of the disease" (? advancing stage), when depression is not noticeable and the septic intoxication is not severe, even when the quantity of pus is large, circumscribed or not circumscribed, he removes the appendix; but that he does not if the patient is apparently overwhelmed by septic intoxication, for in such cases he contents himself by making a simple incision in the abdomen and relieving the pus tension by insertion of a drainage tube, without irrigation, sponging, or manipulation of the tissues. He removes the appendix in nine out of ten cases operated on in the second stage.

It is difficult to know quite what to make of this. If adhesions have formed which circumscribe the pus it is very rarely possible to get at the root of the appendix without separating them, and perhaps separating them widely, though the extremity of the appendix may lie temptingly free in the abscess cavity. It is clear he would not destroy such "life-saving" adhesions if there were clinical signs of toxæmia present; he would simply incise and put in a drainage tube. Whether he would in other cases I must say he does not make clear. If the separation of such adhesions in the presence of virulent pus would be so disastrous in cases in which grave toxæmia is present, because it may increase it, surely it might lead to such a condition in other cases.

Murphy recommends that in all the suppurating cases the patient should be kept in the sitting position for some days after the operation to favour the drainage. In cases of general peritonitis he advises the administration of from eight to twenty-four pints of saline solution per rectum in twenty-four hours. He says "the saline must be allowed to sup in, the tube remaining constantly in position," and he gives no water by the mouth in these cases. He administers calomel, the action of which one would expect rather to interfere with the retention of the saline per rectum. Mr. Bernard is a strong advocate[1] of the **transfusion of a large quantity of saline solution in cases of general peritonitis** by the subcutaneous method. He says the treatment was suggested to him by one of Professor Kocher's assistants, as having proved of great value in Kocher's clinic, and that he and some of his surgical colleagues at the London Hospital have been much encouraged in operating on cases of general septic peritonitis by its adoption in the after-treatment, as so many more now recover. He transfuses as much as fifteen pints in the twenty-four hours in an adult by means of two needles inserted into the subcutaneous tissue of the thigh. The two needles are connected by a rubber tube (with glass tube connection) with a receptacle holding sterile saline of the strength of a teaspoonful to the pint. He uses the tube as a siphon, and directs that the vessel holding the saline solution should be one foot above the needles, and not higher than this, or pain would be caused. The vessel holding the saline and all the apparatus must, of course, be sterilised, and also the skin of the patient. As this treatment has to be carried out in patients desperately ill, no doubt at times the antiseptic precautions may not be perfect, and he has had himself some cases of suppuration where the needle was inserted; but, as he says, these patients were so bad

[1] *Clinical Journal*, 1903, xxii. 380.

at the time of operation that they were fortunate to have lived long enough to develop such abscesses. I must say in one case in which I tried the method myself after operation for intestinal obstruction extensive sloughing of the skin and cellular tissue of the axilla followed, though the vessel containing the saline was not more than six inches above the needle, the fluid was warm saline of the strength mentioned, and every precaution to keep the apparatus and skin aseptic was taken. The needle was certainly large, as I could not get any amount to run in through a small needle which I first inserted. Mr. Bernard writes of the result of this "massive" saline transfusion in general septic peritonitis that "the effect is really very wonderful; the whole face of the patient fills out, the rings round the eyes diminish or may even disappear, the tongue becomes larger and moist as the secretion of saliva is once more established, the pulse is greatly improved and becomes slower and stronger." The transfusion is repeated day by day as occasion demands, unless the vomiting ceases and the patient can drink freely, or the rectum will absorb a pint six times a day. In explanation of the beneficial action of this saline transfusion, he draws attention to the great shortage of fluid in the body in these cases, as owing to the vomiting no fluid has been taken, or if taken absorbed from the stomach, but that during that time the patient has expended his stock of fluid in supplying the excretion of urine, sweat and copious vomiting; and he says patients suffering from general peritonitis lose weight very rapidly from loss of water, and he points to the shrivelled tongue, depressed eyes, and the drying up of the secretion of saliva in the mouth as results of this loss. As he says, they cannot drink to replace this water, and, unlike Murphy, he regards the purgation produced by the frequent administration of calomel, which he regards as an important element in the treatment, as likely to very much interfere with the absorption of saline solution allowed to run into the rectum, and hence he advises the use of the subcutaneous method. And, he points out, the more fluid we can introduce into the vessels the less the tendency will be to absorption of toxic fluid from the peritoneum; and the toxine already absorbed, or that may continue to be absorbed, will be diluted.

As it is now fourteen years since Coley commenced **the treatment of cases of inoperable sarcoma with injections of the erysipelas streptococcus toxines,** his paper in the *American Journal of Medical Sciences*, March, 1906, on his late results, is of considerable interest. He draws attention to the fact that the results necessarily vary greatly with the preparation of the toxines used, and says that many of the preparations put upon the market have been so weak as to produce scarcely any reaction. Parke, Davis & Co., he says, have very nearly brought their standard up to that of the toxine prepared for his own use. As is, of course, well known, his preparation consists of mixed toxines of erysipelas streptococcus and bacillus prodigiosus. With regard to indication as to continuance of the treatment, he says "in successful cases a marked improvement is usually noted in from one to four weeks; and if no improvement has occurred at the end of four weeks of vigorous treatment the chances are that none will occur, and little is to be hoped for, more than a retardation of growth." He describes the technique of administration again in this recent paper, and says that the long-continued use of the toxine does no harm, and mentions one case in which for frequently-recurring sarcoma the toxines were administered, with occasional intervals, for four years with a successful result. When used after removal of sarcoma to try and prevent recurrence he recommends the use of a small dose which will only produce slight symptoms, and not a rigor. Coley still considers that spindle-celled sarcoma is the kind which is most likely to be cured by this method. He says that he has altered his opinion as to the use of the toxines in cases with metastases, and would now employ it, and he refers to some successful cases of this class.

With regard to the use of the toxines as a prophylactic after primary operations for sarcoma and carcinoma, Coley says: "Up to the present I have not felt that the number of observations were sufficient to warrant me in advising such treatment as a routine measure, but I am gradually becoming convinced of the wisdom of so doing." He then gives a short account of eleven cases in which he has used the toxines in this way. Two were cases of carcinoma, and he says: "These cases show that the toxines do have a marked inhibitory action even in carcinoma, and although this is rarely curative, I believe it sufficient to justify the routine use of the toxines after all primary operations for carcinoma as well as sarcoma." Recurrence of malignant growths after removal is always so uncertain, that the number of cases would have to be very large, and the nature of the growth very distinctly malignant, to convince us of the wisdom of using the toxines as a prophylactic from results obtained; but, as Coley points out, it is evident that a toxine which when injected into the body at a distance from the tumour may cause its disappearance, may destroy the microscopic tumour elements in the region of operation or elsewhere in the body, which would otherwise develop into secondary growths. It is interesting to note that Coley regarded the toxines as exercising a distinct restraining influence on these two cases of carcinoma, for until the publication of this paper it seemed that he regarded sarcoma alone as influenced by the use of the toxines. In a list which he gives of cases in which an attack of erysipelas occurred during the growth of a malignant tumour (page 385) is one case of cancer of the breast, and two cases of epithelioma of the face (probably rodent ulcers), which disappeared after an attack of erysipelas. Rodent ulcer can now, of course, be successfully treated by X-rays, and toxines of erysipelas would be very rarely required.

In twenty-six cases of Coley's treated by injections of the mixed toxines the patients were alive and well from three to thirteen years after treatment. All these cases were, he says, "beyond operation, and even absolutely hopeless, as regards treatment of any hitherto known method." It is very important, as Coley says, to note that in five cases in which the tumours disappeared after injection of the toxines, a recurrence took place and finally proved fatal. In one of these recurrent cases the patient had remained well for eight years; in one three and a quarter years; in one two and a half years; in one seven months; and in one six months. Whether the toxines were used again for the final recurrences or not Coley does not say. If they had once caused the tumour to disappear it would be very remarkable if they were not. But at any rate, as Coley says, these cases furnish absolute proof of the correctness of the diagnosis, and refute the early objections brought against the method that the successful cases were probably examples of errors in diagnosis.

It is not very rare for a large mass within the abdomen to be diagnosed as a malignant tumour and then for the mass to disappear, so that it is important to note how many of Coley's successful cases were of this nature. I find three in his list of eighty-six; but in one of these the mass recurred, which would be evidence in favour of its being a malignant tumour.

Coley has also collected the final results of other surgeons who have published successful cases. Most of these surgeons are Americans, but the list includes cases of Battle and Mansell Moullin from this country. Out of sixty cases twenty-seven were well three to twelve years after the disappearance of the tumour. No less than nineteen included in the sixty were intra-abdominal tumours. That such cases should be said to show the structure of round or spindle-celled sarcoma under the microscope cannot differentiate them from simple granulation tissue. To these sixty I may add a case of my own, a young woman with periosteal sarcoma attached to the side and base of the skull in the zygomatic region. It was explored and examined microscopically, and presented the typical appearance of periosteal sarcoma. There was nothing to suggest syphilis. It ceased to grow under the treatment with the toxines, and all that remained of it was a bony lump on the skull. I have also had several unsuccessful cases, but in these the use of the toxines did no harm, though the symptoms of a sharp reaction are distressing for some hours. It would be very instructive if Coley had collected as many of the fatal cases after the use of the toxines as he could. Perhaps he has mentioned all he knows of, but he does not say so. He refers to two deaths from septicæmia after sloughing of the tumours in cases treated by the toxines. But sloughing or even suppuration in the tumour from the injections of the toxines seems to be a rare event.

Coley considers the use of the toxines before resorting to amputation in cases of sarcoma of the extremities. He has collected twelve cases of sarcoma of the extremities, three personal cases, and nine reported by other surgeons in which the use of the toxines rendered amputation unnecessary. Of these eight were free from recurrence from three to six years. In all the cases but one the tumour was of bony origin, and in all amputation had been seriously considered; but it seemed justifiable to give the toxines a trial before resorting to amputation. By means of resection of bone, no doubt amputation might also have been avoided, a method of treatment now employed by some of the German surgeons even in periosteal sarcoma[1] with no recurrence in some cases when examined several years after.

Many of Coley's successful cases of treatment of sarcoma in various regions, are recorded at some length, and a study of these records are most important, but we must refer readers of this abstract to the original paper for them.

CHARLES A. MORTON.

